# Perception gaps between patients with ulcerative colitis and healthcare professionals: an online survey

**DOI:** 10.1186/1471-230X-12-108

**Published:** 2012-08-15

**Authors:** Stefan Schreiber, Julián Panés, Edouard Louis, Derek Holley, Mandy Buch, Kristine Paridaens

**Affiliations:** 1 Department of Medicine I, University Hospital Schleswig-Holstein, Christian Albrechts University, Kiel, Germany; 2 Department of Gastroenterology, Hospital Clínic of Barcelona, IDIBAPS, CIBERehd, Barcelona, Spain; 3 Department of Gastroenterology, University Hospital of Liège (CHU), Liège University, Liège, Belgium; 4 GfK HealthCare, London, United Kingdom; 5 Shire AG, Eysins, Switzerland

**Keywords:** 5-aminosalicyclic acid, Survey, Physicians, Nurses, Quality of life, Ulcerative colitis

## Abstract

**Background:**

The purpose of this study was to examine the differing perspectives and perceptual gaps relating to ulcerative colitis (UC) symptoms and their management between patients and healthcare professionals (HCPs).

**Methods:**

Structured, cross-sectional, Web-based questionnaires designed to assess a variety of disease indices were completed by adult patients with UC and HCPs involved in the care of patients with UC from Canada, France, Germany, Ireland, Spain, and the United Kingdom.

**Results:**

Surveys were completed by 775 patients, 475 physicians, and 50 nurses. Patient self-reported classification of disease severity revealed generally greater severity (mild, 32%; moderate, 53%) compared with physician and nurse estimates of UC severity among their caseloads (mild, 52% and 49%; moderate, 34% and 37%, respectively). Patients reported that an average of 5.5 (standard deviation, 11.0) flares (self-defined) occurred over the past year, compared with 3.4 and 3.8 flares per year estimated by physicians and nurses. Perceived flare triggers differed between patients (stress ranked first) and HCPs (natural disease course ranked first). Fifty-five percent of patients stated that UC symptoms over the past year had affected their quality of life, while physicians and nurses estimated that 35% to 37% of patients would have a reduced quality of life over the same period. Patients ranked urgency and pain as the most bothersome symptoms, while physicians and nurses ranked urgency and stool frequency highest. About half of patients (47%) defined remission as experiencing no symptoms; by comparison, 62% to 63% of HCPs defined remission as requiring the complete absence of symptoms. HCPs (doctors/nurses in general practice and/or hospital) were regarded by patients as their main source of UC information by 72%; however, 59% reported not arranging regular visits to see their HCPs.

**Conclusions:**

This large survey identified important differences between patients' and HCPs' perceptions of the impact of UC symptoms on patients' lives. Notably, HCPs may underestimate the effect of specific UC symptoms on patients and may fail to recognize issues that are important to patients.

## Background

Ulcerative colitis (UC) is an inflammatory disease of the large bowel currently thought to affect 120 to 200 per 100,000 people throughout the Western world [1]. Manifesting clinically as a diverse range of relapsing–remitting gastrointestinal and systemic symptoms, sufferers can find the management of their disease challenging, often resulting in reduced health-related quality of life and poor life satisfaction [2]. Although a number of differing treatments and management strategies are available [3-5], previous studies (predominantly based in the United States) have shown that patients’ and physicians’ perceptions and opinions regarding UC, its management, and specific therapies often differ [6,7]. In particular, the Ulcerative Colitis: New Observations on Remission, Management and Lifestyle (UC: NORMAL) survey observed that physicians tended to underestimate the burden of disease on patients, while many patients considered their symptoms/flares to be “normal” [7].

Following on from previous surveys, this study, conducted in 5 European countries and Canada, was designed to elucidate if any differences exist between patients living with UC and the healthcare professionals (HCPs) who provide treatment for such patients in their respective perceptions on living with UC. Notably, this study also explored the viewpoints of specialist nurses whose perspectives, despite regular involvement in the long-term management of patients in many countries, are not often examined in this kind of survey.

## Methods

### Respondents

Patients who participated in this online survey were aged ≥18 years and had a previous formal clinical diagnosis of UC (any severity). Patients were excluded if they had previously undergone a full or partial colectomy. Physicians were considered eligible for this study if they were experienced gastroenterologists or internal medicine (IM) physicians with a special interest in gastrointestinal medicine and were actively involved in the treatment and management of patients with UC. Nurses were considered eligible if they were specialists in inflammatory bowel disease (IBD) or gastroenterology and were actively managing patients with UC; all nurses were required to see a minimum of 20 patients with UC per month. Both physician and nurse respondents were excluded if: total clinical care/practice (not just on UC) occupied <50% of their normal workload; they did not personally consult patients with UC; they were engaged as a consultant or advisor to the pharmaceutical industry; or if they had qualified to practice within the last 2 (nurses) or 3 (physicians) years.

All respondents provided their consent before completing the questionnaire and were remunerated on behalf of the investigators by the sponsor (Shire Pharmaceuticals LLC, Wayne, PA, USA) for their participation in the survey.

### Study design

This was an Internet-based survey of patients with UC and practicing HCPs (gastroenterologists, IM physicians specializing in gastrointestinal medicine and IBD/gastroenterology nurses) from 5 European countries (France, Germany, the Republic of Ireland, Spain, the United Kingdom) and Canada. All HCPs were involved in the treatment of patients with UC, although they were not required to be directly linked to the patients enrolled in this study.

All respondents were pre-identified from patient and physician/nurse access panels or via custom “phone-to-Web” recruitment, and then rescreened using the previously described criteria to confirm eligibility. For those respondents whose e-mail addresses were included in the access panel/sample lists, e-mail invitations including a link to the online survey were sent without any prior phone or personal contact. In cases where the panel or sample list included only phone numbers, respondents were called and asked for their e-mail addresses; a link to the respective survey was then sent while the respondent was on the phone (phone-to-Web recruitment). All sampling was conducted as randomly as possible with full geographical dispersal within each of the countries. Patient advocacy groups and organizations were not used to recruit patients, so as to avoid potential bias by over-sampling patients who were likely to be more aware of their condition and perhaps more actively engaged with UC management. The market research conducted was fully compliant with the British Healthcare Business Intelligence Association (BHBIA) Legal and Ethical Framework for Healthcare Market Research, which provides best practice guidelines for healthcare market research within an up to date legal and ethical framework. According to these guidelines, market research “falls outside the remit of the Research Governance Framework and does not require Research Ethics Committee approval” [8]. Local market research guidelines were taken into consideration in the countries outside of the United Kingdom where patients were surveyed.

### Survey tool

To record respondents’ opinions and perceptions of UC and its management, three structured, cross-sectional, self-administered, computer-aided Internet-based questionnaires were prepared by GfK HealthCare (London, United Kingdom), a market research company, with input from the study sponsor (Shire Pharmaceuticals LLC). The questionnaires were developed for patients, physicians, and nurses, respectively, based primarily on the previously reported US UC: NORMAL Internet survey questionnaire [7,9]. The questionnaires were piloted on a mix of 13 physicians, nurses, and patient respondents in the UK and Canada in May of 2010. The survey design was reviewed and finalized by a purpose-assembled working group of UC experts, including SS, JP and EL.

The three survey questionnaires included questions to assess multiple aspects of UC and its management including: symptoms experienced; perceptions of remission; the impact of UC on patient quality of life; and relationships between treating physicians and patients (Additional file 1, Additional file 2 and Additional file 3). Definitions for specific terms within the surveys, such as “normal” or “flare” were not provided to respondents, but were defined by respondents themselves based on their own perceptions. Symptom scores were based on a scale of 0 to 10, with higher scores relating to greater severity. Physicians and nurses were asked to evaluate UC in the context of all patients with UC that they were currently treating.

The patient, physician, and nurse questionnaires comprised 64, 44, and 49 main questions each and were estimated to take 30, 24, and 30 minutes to complete, respectively. Before being administered to the whole study population, all three questionnaires were piloted and tested in two central locations by 13 HCP and patient respondents to ensure that items were correctly understood and key issues covered appropriately. Seven respondents tested the pilot questionnaires on May 13, 2010, in the United Kingdom; six more tested them on May 17, 2010, in Canada. No major modifications were made to any of the questionnaires after the pilot testing; however, minor changes were made to the wording of some questions to ensure they would be understood correctly. The surveys were translated into the native language for each country.

### Analysis

The primary aim of this study was to assess patients’ and HCPs’ differing perceptions of UC and its associated treatment. The results of a separate analysis of the survey data, which aimed to explore national differences in patients’ experiences, expectations, and beliefs about UC and its management, will be published elsewhere.

Prior to analysis, all responses were checked by GfK HealthCare for sense, quality, consistency, and reliability. No quality issues were identified for this study. With regard to statistical accuracy, for patients, a range of ±3.3% to ±7.6% (at 95% confidence interval [CI] limits; survey percentage, 5%-50%) was calculated at the individual country level, assuming 150 respondents per country; likewise, a statistical accuracy range of ±5.9% to ±13.6% (at 95% CI limits) was determined if 50 patient respondents per country were assumed. For physicians, a statistical accuracy range of ±4.1% to ±9.5% was calculated at the individual country level, assuming 100 respondents per country. Comparisons drawn between groups (eg, patients and physicians) are descriptive and no statistical tests were performed.

## Results

### Respondents

Surveys were completed by 775 patients, 475 physicians, and 50 nurses between June 10, 2010, and August 20, 2010, in Canada, Germany, Ireland, Spain, and the United Kingdom, and between January 20, 2010, and February 24, 2011, in France. Among patients who completed questionnaires, 150 each were submitted by patients in France, Germany, Spain, and the United Kingdom, 125 were submitted in Canada, and 50 in Ireland. Patient response rates (RR) were not calculated, as different methods of recruitment were used across countries. Completed surveys were submitted by 100 physicians each in France (RR 18%), Germany (RR 23%), Spain (RR 17%), and the United Kingdom (RR 19%), 60 in Canada (all gastroenterologists; RR 6%), and 15 in Ireland (RR 12%). Fifty nurses (RR 15%) completed surveys, all of whom were based in the United Kingdom.

Demographic and baseline data for all patient, physician, and nurse respondents are shown in Table 1. A majority of patients were female and approximately half were married; 59% were in full- or part-time employment. Comorbidities present in ≥10% of patients included chronic back pain, migraine, arthritis, depression, and asthma. Approximately half of patients were symptomatic at the time of the survey and just over three-quarters were currently taking prescription medication for their UC; 5-aminosalicylic acid (5-ASA) was the most commonly prescribed UC medication (75%), followed by corticosteroids (32%), immunosuppressants (24%), and biological therapies (anti-tumor necrosis factor agents or other; 15%). Overall, physicians surveyed (gastroenterologists, 82%; internal medicine physicians, 18%) tended to be male and were working predominantly in city-based, public teaching hospitals. Surveyed physicians devoted a mean (standard deviation [SD]) of 87% (11%) of their working day to clinical practice, and saw a mean (SD) of 35 (33) patients with UC in a typical month. Nurses were predominately female (92%), most were clinical nurse specialists (84%), and a mean (SD) of 81% (14%) of their day was devoted to direct patient care.

**Table 1:** Demographic and Baseline Characteristics of Patients and HCPs

**Characteristic**	
***Patients***	
N	775
Male, %	37
Median age, year (SD)	44.6 (15.1)
National patient organization member, %	10
Marital status, %	
Single, no partner	25
Single, with partner	26
Married	49
Employment, %	
Full-time	43
Part-time	16
Comorbidity ≥10 % incidence, %	
Chronic back pain	15
Migraine	14
Arthritis	13
Depression	12
Asthma	10
UC status, %	
In flare	11
Mildly symptomatic	43
In remission	46
Currently receiving UC therapy, %	76
Type of UC therapy, %*	
5-ASA/aminosalicylate	75
Monotherapy	43
Corticosteroids	32
Immune therapy^†^	24
Antibiotics	8
Biological therapy	15
Other	9
***Physicians***	
N	475
Male, %	82
Mean qualification year (SD)	1992 (9.1)
Primary specialty, n	
Gastroenterology	82
Internal medicine	18
Lower GI/IBD specialist, %	68
Office- or hospital-based, %	
Office	19
Hospital	64
Equal	17
Location, %	
City center	71
Suburban	23
Rural	6
Teaching hospital, %	67
Type of hospital, %	
Public	89
Private	11
Mean % of working day devoted to clinical practice (SD)	87.4 (11.2)
Mean number of patients with UC seen in typical month, n (SD)	35.2 (33.0)
***Nurses***	
N	50
Male, %	8
Mean year of qualification (SD)	1992 (7.5)
Mean year becoming IBD Nurse (SD)	2003 (3.8)
Type of nurse	
Nurse practitioner	10
Nurse prescriber	6
Clinical nurse specialist	84
Location, %	
City center	56
Suburban	40
Rural	4
Teaching hospital, %	80
Mean % of working day devoted to direct patient care (SD)	80.8 (14.1)
Mean number of patients with UC seen in typical month, n (SD)	66.6 (40.2)
Percentage of nurses who make treatment decisions for patients with UC	86
Percentage of nurses who are qualified nurse prescriber	44
Percentage of nurses who write 5-ASA prescriptions for mild-to-moderate UC	77
Mean number of 5-ASA prescriptions written per month (SD)	21.4 (15.9)

HCP, healthcare professional; SD, standard deviation; UC, ulcerative colitis; 5-ASA, 5-aminosalicylic acid; GI, gastrointestinal; IBD, inflammatory bowel disease.

*All patients currently taking prescription medication for their UC.

^†^Immunomodulator or immunosuppressant.

### Perceptions of UC symptoms

**Figure 1:**
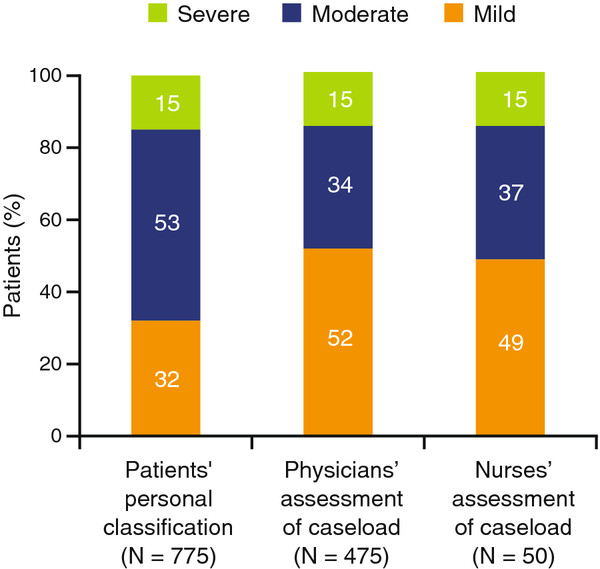
**UC severity ratings.** Patients’ self-reported classification of disease severity: patients were asked how they would personally describe the severity of their UC overall, regardless of how their doctor described it. Physicians’ and nurses’ assessment of symptom severity among their caseloads: physicians and nurses were asked what percentage of their current UC patients had mild, moderate, or severe disease. Data may not add up to 100 % due to rounding. UC, ulcerative colitis.

Physicians assessed UC among their caseloads to be less severe compared with the overall profile of UC patients’ self-reported disease severity (Figure 1). Overall, 32%, 53%, and 15% of patients described themselves as having mild, moderate, and severe UC, respectively. In contrast, physicians estimated their UC caseloads to be comprised of a mean (SD) of 52% (19%) mild cases, 34% (14%) moderate cases, and 15% (12%) severe cases, respectively. Nurses’ estimates of their UC caseloads were similar to those of physicians: mean (SD) of 49% (22%) mild, 37% (17%) moderate, and 15% (9%) severe. Patients were not paired with their HCP; thus it is not known if HCP and patient descriptions of UC severity agreed for any individual patient. When patients were questioned about how they felt their own physician perceived their disease, 8% were unsure. Of those who had a clear opinion, the resulting profile of patients’ perception of their physicians’ severity ratings was almost identical to that of patients’ self-reported UC severity (mild, 33% vs 32%; moderate, 53% vs 53%; severe, 14% vs 15%; respectively).

**Figure 2:**
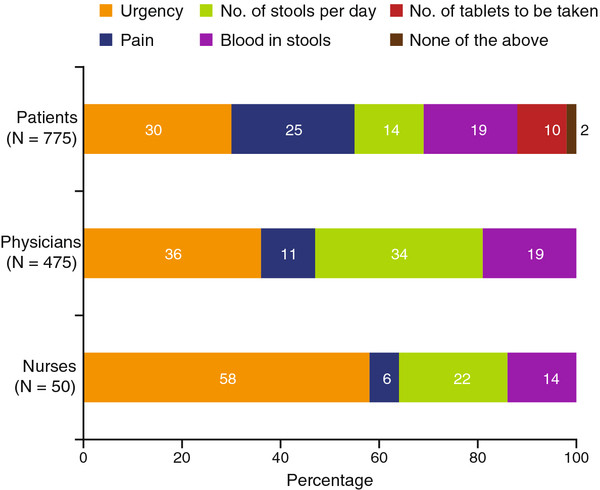
**Most bothersome factor for UC patients, as assessed by patients, physicians, and nurses.** Patients were asked which one of the following bothered them the most about their UC: urgency, pain, number of stools per day, blood in your stools, number of tablets to be taken, or none of the above. Physicians and nurses were asked which one of the same choices they thought bothered their UC patients the most. Data may not add up to 100 % due to rounding. UC, ulcerative colitis.

There were also differences in perception among patients, physicians, and nurses in terms of what was most bothersome to patients about their UC (Figure 2). Urgency and pain were most frequently ranked as the most bothersome symptom by patients (30% and 25%, respectively). For physicians and nurses, urgency (36% and 58%, respectively) and stool frequency (34% and 22%, respectively) were most frequently ranked as most bothersome. Pain was ranked as most bothersome by only 11% and 6% of physicians and nurses, respectively.

### Beliefs and perceptions surrounding UC flares

Physician and nurse estimates of the typical mean (SD) number of flares per year experienced by a patient with UC were lower (3.4 [3.0] and 3.8 [8.0], respectively) than patients’ estimates of the mean (SD) number of UC flares that they experienced (5.5 [11.0]). Approximately two-thirds of patients (68%) considered the number of flares they had experienced during the past 12 months to be normal; these patients reported a mean (SD) of 3.9 (5.2) flares, while those who considered the number of flares they had experienced to be abnormal reported a mean (SD) of 5.5 (8.1) flares. Out of these 5.5 flares/year experienced, patients reported discussing a mean (SD) of 4.2 (7.1) of them with their primary HCP. The most common reasons patients gave for not discussing flares with their doctor or nurse were: “some attacks were milder/not too bad/cleared up quickly” (23%), “dealt with/managed it on my own/learned how to deal with it/self management” (21%) and “settled/managed it on my own using (prescribed/self) medication” (17%).

**Figure 3:**
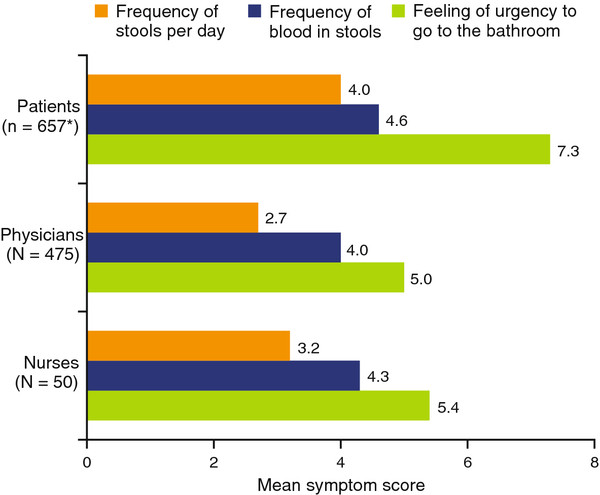
**Mean symptom ratings during a flare in mild-to-moderate UC, as assessed by patients, physicians, and nurses.** Patients with mild-to-moderate disease were asked to rate each of the following to describe a typical flare of UC on a continuous rating scale: frequency of stools per day, frequency of blood in stools, and feeling of urgency to go to the bathroom. Physicians and nurses were asked to rate the same choices according to how they would define a typical flare experienced by a mild-to-moderate patient. Ratings were transformed to a 10-point scale; higher scores indicated greater symptom severity. UC, ulcerative colitis

**Figure 4:**
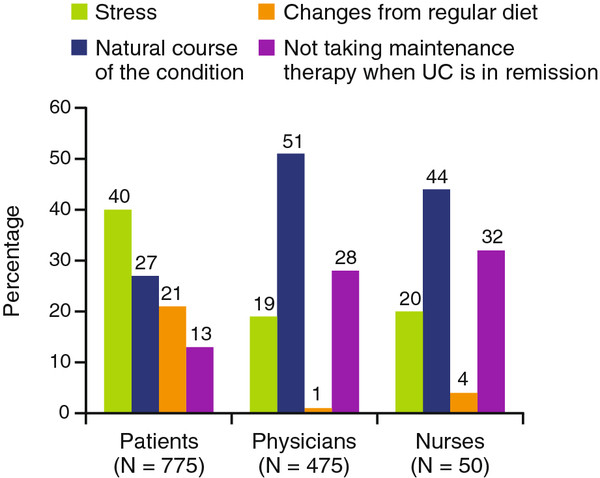
**Most likely cause of UC flares, as ranked by patients, physicians, and nurses.** Patients, physicians, and nurses were asked, based on their own viewpoint, to place the following four causes of UC flare in rank order: stress, natural course of the condition, changes from regular diet, and not taking maintenance therapy when UC is in remission. Data may not add up to 100 % due to rounding. UC, ulcerative colitis

When asked to rate symptom severity during a flare on a continuous rating scale, patients’ ratings were higher (more severe) than those estimated by HCPs on frequency of stools, frequency of blood in stools, and urgency (Figure 3). When asked to rank-order the most common causes of flare among “natural course of the condition,” “changes from regular diet,” “stress,” and “not taking preventative therapy,” the rank orders differed between patients and both physicians and nurses (Figure 4). Compared with patients, a higher percentage of physicians and nurses, respectively, ranked “natural disease course” followed by “not taking preventative therapy” as the most common causes. By contrast, patients ranked “stress” as the most likely trigger of UC flares.

### Perceptions of remission

Nearly half (47%) of all patients surveyed stated that remission realistically involved “experiencing no symptoms and feeling similar to how I did before I developed UC”; all but 2% of the remaining patients believed that they could be in remission while still experiencing some symptoms. According to their own estimates, patients who stated that remission involved “living without symptoms” experienced a lower average number of flares during the past 12 months compared with those who stated that remission involved “living with some symptoms” (4.2 vs 8.2 flares per year, respectively).

Compared with patient expectations of remission, an even greater percentage of physicians (63%) and nurses (62%) agreed that remission required the “complete absence of symptoms” (Table 2). However, the physician survey probed deeper into HCP considerations of remission than the patient survey. Both physicians and nurses favored “normalized” endoscopy and quality of life, respectively, rather than “improved” endoscopy and quality of life as requirements of UC remission. The majority of physicians and nurses considered patient satisfaction with outcome, an absence of inflammatory markers, and becoming steroid-free as necessary requirements when defining remission (Table 2). Approximately 4 in 10 physicians and nurses recognized that patient definitions of remission were less stringent than their own, compared with nearly 2 in 10 HCPs who believed that patient definitions of remission were more stringent.

**Table 2:** Necessary Personal Requirements of Remission, As Selected by Physicians and Nurses

**Remission requirements (%)**	**Physicians**	**Nurses**
Reduced symptoms	36	38
OR		
Complete absence of symptoms	63	62
Normalized endoscopy score	50	64
OR		
Improved endoscopy score	45	34
Quality of life–normalized	64	62
OR		
Quality of life–improved	35	38
Patient appears content with treatment outcome	72	80
No laboratory indicators of inflammation, such as C-reactive protein	69	54
Absence of steroids	60	70

Physicians and nurses were asked, based on their personal definitions, to select the requirements for remission from three paired options (symptoms, endoscopy score, quality of life) and three unpaired options. Respondents could only select one option for each of the paired options, but could also select any or none of the remaining three options.

### Burden of disease and perceived disease control

**Figure 5:**
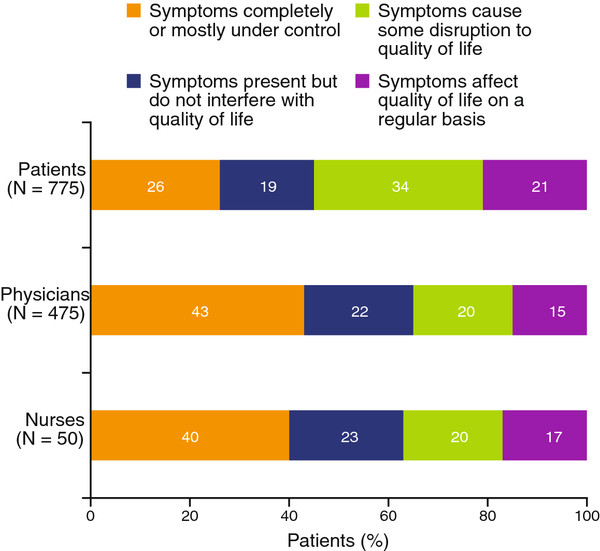
**Disease control over the past 12 months, as assessed by patients, physicians, and nurses.** Patients were asked which one of the following statements best described how effectively their UC had been controlled over the past 12 months: (1) my symptoms were completely or mostly under control; (2) my symptoms were present but did not interfere with my quality of life; (3) my symptoms caused some disruption to my quality of life; or (4) my symptoms affected my quality of life on a regular basis. Physicians and nurses were asked, in terms of how effectively their UC had been controlled over the last 12 months, approximately what percentage of your mild-to-moderate UC patients fell into each of the above categories. Data may not add up to 100 % due to rounding**.** UC, ulcerative colitis

When asked to determine how well UC was controlled over the past year, 55% of patients responded that symptoms of UC caused at least some disruption to their quality of life; physicians and nurses perceived UC as affecting quality of life in only 35% to 37% patients, respectively (Figure 5). In addition, a lower percentage of patients (26%) considered UC symptoms to be completely or mostly under control compared with the perceptions of physicians (43%) or nurses (40%). A large proportion of patients strongly agreed (49%) or somewhat agreed (38%) that they were worried about the long-term health effects of having UC.

### Beliefs and perceptions about treatment with 5-ASA

Three-quarters of patients (75%) were currently receiving a 5-ASA for the treatment of their UC; 58% of those using 5-ASA were taking it as monotherapy. The overall percentage of patient self-reported adherence with prescribed oral 5-ASA therapy during their most recent period of remission (67%) was slightly higher than physicians’ (62%) and nurses’ (57%) estimates of adherence among their caseloads. Among the non-adherent patients, 47% had tried not taking their medication at all for a period of time and 53% tried taking less than what was prescribed. Over a third of non-adherent patients (38%) either did not reveal (20%) or under-reported (18%) the extent of their non-adherence to their HCP. When asked to think about the last 7 days, 53% of patients stated that they had taken less than their prescribed dose of 5-ASA, with 5% not taking any medication at all.

The majority (78%) of patients were “very” or “somewhat” satisfied with 5-ASA treatment, consistent with physicians’ perception of patient satisfaction (physicians estimated that 90% of their patients were “very” or “somewhat” satisfied). Among dissatisfied patients, only 35% discussed their dissatisfaction with their doctor. Ninety-one percent of patients currently receiving oral 5-ASA therapy reported that they would be willing to switch to a once-daily formulation of 5-ASA during disease remission if their doctor suggested it. However, 30% of physicians said that they were unlikely to offer a once-daily oral 5-ASA to a patient in remission currently taking oral 5-ASA twice daily.

### Relationship between patients and HCPs

Almost three-quarters of patients (72%) ranked doctors and/or nurses at their general practice/surgery or hospital among their top three sources of information about UC and the different treatment options available, and 45% reported UC-related Web sites as another top source. Despite this, 56% stated that they did not see their doctor or specialist nurse on a regular basis, and another 3% stated that they never visit their doctor or specialist nurse. The remaining patients stated that they visited their main HCP when feeling unwell or having a flare-up (29%), or only if experiencing a serious flare (27%). Notably, 64% of patients expressed that they had never taken the initiative to ask their doctor about new medications and treatment options for UC.

While 69% of patients considered themselves to be completely open with their doctor about their UC during consultations, another 21% considered themselves open only if carefully questioned; the remaining patients admitted keeping information from their physician. Male and female patients appeared to be equally open to discussion of their UC during healthcare visits, with 68% and 69%, respectively, describing themselves as being completely open. Among the 40% of physician respondents who shared patient management with nurses, accessibility to patients (70%) and having more time to spend with patients (44%) were ranked as the top strengths of the care provided by nurses, suggesting that physicians perceived nurses as a potential resource for improving communication with patients.

## Discussion

This survey provides insight into the differing experiences and perceptions of patients with UC and HCPs involved in the management of such patients across Western Europe and Canada. To our knowledge, this is the first international study in UC to compare the views and beliefs of patients, physicians, and specialist nurses.

A higher percentage of patients reported that UC symptoms adversely affected their quality of life compared with the percentage of patients perceived by HCPs to have quality of life disruptions. Furthermore, a majority of patients assessed their own symptoms to be moderate in severity, while HCPs estimated the majority of their patients as having UC of mild severity. These results suggest that physicians and nurses may underestimate patients’ symptom burden. Our data also demonstrated a gap among patients and HCPs with regard to what bothers patients most about their UC. Whereas patients most frequently ranked urgency, pain, and bloody stools as bothersome factors relating to their UC, HCPs perceived urgency and stool frequency as being most bothersome. This discordance suggests that physicians and nurses may need to be more aware of patients’ feelings in regards to level of pain and bloody stools; it should be noted that a relatively high prevalence of chronic pain as a patient comorbidity at baseline (15%) may have influenced the results.

Physicians’ and nurses’ estimates of the annual incidence of flares experienced by a patient with UC were lower than the patient-reported number of UC flares. A similar finding was reported from the UC: NORMAL survey [7], suggesting either a potential underestimation of patients’ symptoms by HCPs, or a misinterpretation of symptoms by patients. In addition, patients in this study only discussed approximately three-quarters of their experienced flares (self-defined) with their HCP. This patient-physician gap suggests a need for more open lines of communication and improved patient education on symptom evaluation, so that patients will be better able to define when a flare is occurring.

Approximately half of patients defined remission as the complete absence of symptoms compared with 62% to 63% of nurses and physicians, respectively. This discrepancy is not unexpected given that there is no widely agreed upon definition of UC remission. At present, discussions are unfolding among HCPs about how best to define remission; definitions in clinical trials may be less stringent (ie, some UC symptoms are acceptable) compared with the expectations of clinicians in daily practice noted in this survey. The initiation of a discussion among patients, HCPs, and regulatory authorities on modifying endpoints for new UC medications being developed would be helpful.

Patients were more likely than HCPs to rank stress and diet as the most likely triggers of flares, whereas HCPs were more likely to rank the natural disease course and not taking maintenance therapy as the most likely causes. It may be the case that patients are unaware of the relapsing–remitting nature of the disease [10], and the importance of maintenance therapy during quiescent periods to reduce the probability of a disease flare [11-13]. If patients are not regularly taking maintenance therapy, flare recurrence is more likely [11,13,14], and may subsequently be attributed to causes such as stress and diet; therefore, patient education on UC disease course and medication adherence is critical. It is also possible that patients may have misinterpreted symptoms as UC flare, instead of symptoms of irritable bowel syndrome (IBS). Both conditions share some common symptoms such as pain and alteration of bowel habits [15], in part because of the role of gut microbiota in the pathogenesis of both UC and IBS. Therefore, a better understanding and distinction of UC versus IBS symptoms is important for proper management of UC.

Patients’ estimates of adherence with current oral 5-ASA therapy during their most recent period of remission were broadly similar to HCPs’ estimates. However, patient-estimated adherence rates over the past 7 days were lower. Similar to rates observed in previous observational data [6,7,11,12,14,16-20], these low adherence rates are troublesome, given that poor adherence with 5-ASA therapy is linked to an increased risk of flare and higher medical costs [11-13,21]. In turn, increased disease activity can also result in deterioration of patient quality of life [2,22-24]. Effective patient–physician communication and collaboration has been shown to improve adherence in other chronic diseases [25,26], and may improve adherence rates for patients with UC.

About 20% of patients reported being dissatisfied with 5-ASA treatment while physicians perceived that only 10% of patients were dissatisfied. However, only about one-third of dissatisfied patients reported having discussed their concerns with their doctor. Thus, more direct questioning from HCPs may help patients be more forthcoming. Furthermore, although the large majority of patients (72%) saw their doctor or specialist nurse in general practice and/or hospital as the principal source of information about their UC and its treatment, most (59%) did not arrange regular visits. This lack of regular contact between patients and their HCP may account, in part, for the discrepancies in perception of the impact of UC.

One unique aspect of this survey is that it compares the perception of nurses treating UC with physicians. Given that the sample of nurse specialists was limited to the United Kingdom, it may be of benefit to specifically compare how the responses of UK nurses compared to those of UK physicians. Results from physicians and patients by country are presented in a separate analysis [27], although nurses were not included in that analysis. Briefly, for caseload severity, effect of UC on quality of life, and adherence to 5-ASA treatment, estimates of UK nurses and UK physicians were nearly identical (within 5 percentage points). The rank order of which UC symptoms were most bothersome to patients was the same for UK nurses and UK physicians (urgency, number of stools, blood in stools, and pain). However, UK nurse estimates of flares experienced per year were higher than UK physician estimates for each severity grade (mild, 1.6 vs 0.9; moderate 3.4 vs 2.4; severe, 6.4 vs 4.4). Although it remains unclear how generalizable the nurse data are across nations, the UK data suggest that responses from nurses are likely to be similar to those of physicians from the same country.

As with all observational studies, this study was subject to a number of limitations. The primary limitation was the fact that patients and HCPs were recruited independently and not matched; thus, it is possible that the patient respondents had a more severe disease profile compared with patients included in physicians’ and nurses’ caseloads. Additionally, patients and HCPs were not given standard definitions of disease severity or flares on which to base their responses. Nonetheless, the current findings are in line with that reported in the UC: NORMAL study [7]; the demographic profile of patient respondents was similar to that reported in previous surveys [7,9,28-30] and within the general UC patient population [28,31-33], suggesting that these results may be applicable to the larger UC population.

## Conclusions

In conclusion, results of this European and Canadian survey demonstrate discordance between patients’ self-perceived experience with UC and HCPs’ (physicians and nurses specializing in gastrointestinal medicine) estimates of UC disease burden. These differences could be addressed through improved communication between patients and their physician or specialist nurse (including more regular visits), and better patient education. As patients with other gastrointestinal chronic diseases may have similar experiences to those surveyed in this study regarding disease treatment and management, the findings reported here may also be more widely applicable.

## Competing interests

Stefan Schreiber has received consultancy and speaker fees from Shire that were not related to this article; has received lecture fees from the Falk Foundation; and has consulted for various companies developing novel biologic agents. Julián Panés has received consultancy fees from Abbott, Bristol-Myers Squibb, Cellerix, Genentech, MSD, Novartis, Palau Pharma, Pfizer, and Roche; has received speaker fees from Abbott, Ferring, MSD, Shire, and Tillotts Pharma; and has received unrestricted research grants from Abbott and MSD. Edouard Louis has received consultancy fees from Schering Plough, Abbott, MSD, Ferring, Shire, Millennium, and UCB; has received research or educational grants from MSD, Schering Plough, Astra Zeneca, Abbott; and has received lecture fees from Abbott, Astra Zeneca, Ferring, MSD, Schering Plough, Falk, Menarini, Chiesi, and Nycomed. Derek Holley and Mandy Buch are employees of GfK HealthCare, which was contracted by Shire to conduct this research. Kristine Paridaens is an employee of Shire.

## Authors’ contributions

Conception and design: all authors. Acquisition of data: DK, MB. Analysis and interpretation of data: all authors. Drafting the article or revising it critically for important intellectual content: All authors. Final approval: all authors

## Pre-publication history

The pre-publication history for this paper can be accessed here:

http://www.biomedcentral.com/1471-230X/12/108/prepub

## Supplementary Material

Additional file 1 Final questionnaire completed by physicians. Click here for file

Additional file 2 Final questionnaire completed by nurse specialists. Click here for file

Additional file 3 Final questionnaire completed by patients. Click here for file
