# Supplementary material for: Perception gaps between patients with ulcerative colitis and healthcare professionals: an online survey
Source: BMC Gastroenterol. 2012 Aug 15;12:108. doi: 10.1186/1471-230X-12-108 (PMC3523079; doi:10.1186/1471-230X-12-108)
Supplement: Additional file 1 — Final questionnaire completed by physicians. [file 1471-230X-12-108-S1.doc]

**Appendix 1. Survey of Physicians Who Treat Ulcerative Colitis**

**SCREENING**

S1. What is your primary medical specialty?

Select one.

- Family or General Practitioner
- Internal Medicine
- Internal Medicine with a GI specialty
- Gastroenterology
- General Surgery
- Colorectal Surgery
- Other

S2. Approximately what percentage of your working day is devoted to clinical practice?

1. % working day

S3. Do you currently work in a consultative or advisory capacity to the pharmaceutical industry or to a particular pharmaceutical company?

Select one.

- Yes
- No

S4. In what year did you first qualify as a medical doctor?

[ ]

S5. Approximately how many patients with **ulcerative colitis** do you personally see in a typical month? Please include both new and existing patients in your answer.

1. Patients

S6. Which one of the following would you classify yourself as?

Select one.

- Special interest in upper GI
- Special interest in lower GI/IBD
- Special interest in liver
- Special interest in pancreas
- Special interest in other
- No special interest

**SECTION 1 – Patient characteristics**

1. What percentage of your UC patients currently have:
2. % Mild UC
3. % Moderate UC
4. % Severe UC
5. What would you consider to be a ‘typical’ number of flare-ups per year experienced by a patient with:

Mild UC [ ] flare-ups

Moderate UC [ ] flare-ups

Severe UC [ ] flare-ups

1. In your clinical practice, how would you define a typical UC flare experienced by a mild to moderate patient?

Please adjust the pointer on each of the 3 scales below to indicate how you define a **typical UC flare** in respect to frequency of stools per day, frequency of blood in stools, and urgency.

**Stools per day**: 0­­­­­­­­­­­­­­­­­­­------------------5----------------------10---------------------15--------------------20

**Blood in stools**: No stools-------------------------------------------------------------------------All stools

**Feeling of urgency**: Weak--------------------------------------------------------------------------------Strong

1. If a universal definition of ‘remission’ in mild to moderate UC were to be developed for use in clinical practice, what do you feel it should include?

Please type in your answer as fully as possible in the box below.

1. In your clinical practice, which level of each of the following typically means ‘remission’ in mild to moderate UC?

Please adjust the pointer on each of the 3 scales below to reflect your **UC when in remission**.

**Stools per day**: 0­­­­­­­­­­­­­­­­­­­--------1--------2-------3--------4--------5--------6--------7--------8--------9------10

**Blood in stools**: No stools-------------------------------------------------------------------------All stools

**Feeling of urgency**: Weak--------------------------------------------------------------------------------Strong

1. Thinking again about your personal definition of remission in mild to moderate UC, which of the following are necessary requirements?

Select all that apply.

- Reduced symptoms
- Complete absence of symptoms
- Normalised endoscopy score
- Improved endoscopy score
- Quality of life - Normalised
- Quality of life - Improved
- Patient appears content with treatment outcome
- No laboratory indicators of inflammation, such as CRP
- Absence of steroids
- Other requirements (specify: ___________________________________________________)

1. Do you think your patients’ definitions of remission are any different from yours?

Select one.

- Yes – most patients’ definitions are probably less stringent
- Yes – most patients’ definitions are probably more stringent
- No – most patients’ definitions are probably similar to mine

**SECTION 2 – Medications and 5-ASAs**

1. Approximately what percentage of your mild to moderate UC patients are currently being prescribed:
2. % Oral 5-ASAs
3. % Rectal 5-ASAs
4. % Immunomodulators / immunosuppressants
5. % Corticosteroids
6. % Biologics
7. % Antibiotics
8. % No prescription therapy
9. Please place in rank order, the five treatment goals shown below for treating mild to moderate UC patients in your practice.

Please enter a number between 1 and 5 in each box, with 1 being the most desirable goal and 5 being the least.

1. Controlling flare-ups
2. Mucosal healing
3. Minimising or avoiding complications
4. Maintenance of remission
5. Reducing burden of medication (easier dosing)
6. Overall, how satisfied are you with current 5-ASA medications for UC?

Select one.

- Very satisfied
- Somewhat satisfied
- Not very satisfied
- Not at all satisfied

1. Approximately what percentage of your UC patients do you think are satisfied/happy with their current 5-ASA medication?
2. % patients satisfied/happy
3. Picture a patient in remission, to whom you are prescribing oral mesalazine on a BID basis, and who has not asked you about new or alternative treatments for UC. How likely are you to suggest to him/her that they consider switching from one oral 5-ASA to a once-daily oral 5-ASA?

Select one.

- Very likely
- Somewhat likely
- Not very likely
- Not at all likely

1. In general, how difficult do you think it is for your patients in remission to take their 5-ASA medication every day, at the correct time?

Select one.

- Very difficult
- Somewhat difficult
- Not very difficult
- Not at all difficult

1. When in remission, approximately what percentage of your UC patients who are prescribed 5-ASA medication do you currently believe:
2. % are fully compliant with their 5-ASA medication, as prescribed
3. % have self-reduced their 5-ASA medication (take less tablets or take less frequently)
4. % have stopped taking their 5-ASA medication altogether
5. What do you believe are the **main** reasons that patients are not fully compliant with their 5-ASA medication regimen when in remission?

Select a maximum of two answers.

- They try to cut back on medication when they can
- To avoid possible side-effects
- They forget to take it
- They feel well
- They find it a burden to take all the medication
- They don’t like to be reminded of their UC
- Other reason (specify:________________________________________________________)

**SECTION 3 – Patient relationships and perceptions**

1. Please think about the types of relationships you have with your UC patients. Approximately what percentage fall into each of the following categories?
2. % I make most of the decisions about the patient’s treatment
3. % I have an equal partnership with the patient, where we make decisions together
4. % I advise patients and help them understand their options so they can make the best decisions on their own behalf
5. Based on your experience, how disruptive do you think UC is to a patient’s life?

Select one.

- Very disruptive
- Somewhat disruptive
- Not very disruptive
- Not at all disruptive

1. Which one of the following do you think bothers your UC patients the most?

Select one.

- Blood in their stools
- Number of stools per day
- Urgency
- Pain
- Number of tablets to be taken
- None of the above

1. As best you know or are aware, please estimate the percentage of your mild to moderate UC patients:
2. % for whom UC makes life more stressful
3. % for whom UC makes it difficult to lead a normal life
4. % for whom UC is embarrassing
5. % who worry about the long-term health effects of having UC
6. In terms of how effectively their UC has been controlled over the last 12 months, approximately what percentage of your mild to moderate UC patients fall into each of the following groups:
7. % symptoms are completely or mostly under control
8. % symptoms are present, but do not interfere with their lives
9. % symptoms cause some disruption to patient quality of life
10. % symptoms negatively affect life on a regular basis
11. Please estimate the percentage of your mild to moderate UC patients who…
12. % are sometimes reluctant to tell you about the number of flare-ups they have had between visits
13. % underplay the impact UC is having on their life
14. % are difficult to make sufficiently happy even when their UC is under control
15. % feel their disease is more difficult to live with than you do
16. % are more concerned about the safety of their UC medicines than you are

**SECTION 4 – Overlap with different HCPs**

1. Do you share patient management with any IBD Specialist Nurses?

Select one.

- Yes
- No

1. How many IBD Nurse Specialists do you work with?
2. IBD Nurse Specialists
3. For approximately what percentage of your UC patients experiencing a **flare-up** does each of the following serve as the **first** point of contact:
4. % yourself or another Specialist physician
5. % GP/FP
6. % IBD Nurse Specialist
7. % another healthcare professional
8. And for approximately what percentage of your UC patients **in remission**, does each of the following serve as the **primary** point of contact (for their UC)?
9. % yourself or another Specialist physician
10. % GP/Family doctor
11. % IBD Nurse Specialist
12. % another healthcare professional
13. Which of the following do you consider to be the **top three** strengths of the care provided by IBD Nurse Specialists in gastroenterology/UC?

***Select up to three.***

❑ Being accessible for patients

❑ Managing the patient's overall wellbeing

❑ Giving explanations in a way that patients can understand

❑ Helping patients feel comfortable discussing issues

❑ Recommending additional support resources for patients

❑ Having more time than physicians

❑ Less clinically/scientifically focused and more empathetic

❑ Ability to adjust dosing/prescribe treatment

**SECTION 5 – The Doctor-Patient-Nurse relationship**

1. To what extent do you agree or disagree with each of the following statements?

| Strongly disagree | Somewhat disagree | Somewhat agree | Strongly agree |
| --- | --- | --- | --- |

1. It is primarily my responsibility to make sure my patients feel fully knowledgeable about UC.
2. It is important for me to know how UC affects my patients’ lives.
3. It is my responsibility to help patients with the emotional aspects of having UC.
4. It is my responsibility to ensure patients are aware of new treatment options.
5. For approximately what percentage of your UC patients do IBD Specialist Nurses significantly input to decisions about the patient’s treatment?
6. % UC patients
7. To what extent do you agree or disagree with each of the following statements?

| Strongly disagree | Somewhat disagree | Somewhat agree | Strongly agree |
| --- | --- | --- | --- |

1. IBD Nurse Specialists help to bridge any communication gap between patients and physicians.
2. Patients often do not disclose all their flare-ups with physicians.
3. Patients are often reluctant to discuss the full impact of UC on their lives with physicians.
4. Patients are often reluctant to be fully open about 5-ASA non-adherence with physicians.
5. How comfortable do you think patients are in discussing each of the following with IBD Nurse Specialists compared with physicians?

| Less comfortable with IBD Nurse Specialists | About the same | More comfortable with IBD Nurse Specialists |
| --- | --- | --- |

1. All flare-ups and symptoms that occurred since the last visit
2. The full impact of UC on their lives
3. Difficulties with taking their 5-ASA medication
4. Which of the following do you think is the most frequent reaction of established patients, who have had UC for many years, to a flare-up?

Select one.

- They contact a doctor [or nurse**]**
- They only contact a doctor [or nurse**]** in certain circumstances, such as a particularly bad flare-up
- They manage alone, without seeing a doctor [or nurse**]**

1. Based on your experience, please place in rank order the four causes of UC flare shown below.

Please enter a number between 1 and 4 in each box, with 1 being the most common cause and 4 being the least common.

1. The natural course of the condition
2. Changes from regular diet
3. Stress
4. Not taking preventative therapy when UC is in remission
5. When patients have a flare-up, approximately what percentage, if any, do you think take each of the following treatment actions without first consulting a healthcare professional?
6. % patients who start taking a temporary course of a rectal 5-ASA (e.g. unused doses from when prescribed for a previous flare-up)
7. % patients who start taking a temporary course of oral steroids (e.g. unused doses from when prescribed for a previous flare-up)
8. You mentioned earlier that approximately ***<<from Q14>>*** of your UC patients currently prescribed 5-ASA medication have probably **stopped** taking it altogether when in remission. Of this group of patients, approximately what percentage do you think start taking their oral 5-ASA again when they have a flare-up without first consulting a healthcare professional?
9. % UC patients
10. You mentioned earlier that approximately ***<<from Q14>>*** of your UC patients currently prescribed 5-ASA medication have probably **self-reduced** their dose when in remission. Of this group of patients, approximately what percentage do you think start titrate their dose up again when they have a flare-up without first consulting a healthcare professional?
11. % UC patients
12. Which one of the following do you think best describes your patients’ ‘openness’ with you at consultation?

Select one.

- Most patients are completely open, volunteering all relevant information about their UC symptoms and issues
- Most patients are open, but only after careful questioning
- Most patients probably keep some things from me
- Most patients probably keep many things from me

1. To what extent do you agree or disagree with each of the following statements?

| Strongly disagree | Somewhat disagree | Somewhat agree | Strongly agree |
| --- | --- | --- | --- |

1. I frequently sense my patients are reluctant to tell me of past flare-ups
2. I frequently sense my patients are reluctant to tell me about the impact of UC on their lives
3. My patients are more easily satisfied than I am that their UC is under control

**SECTION 6 – Access to care**

1. **Compared to IBD Nurse Specialists, how much time do you feel you can spend with UC patients?**

Select one.

- **Much less time than an IBD nurse**
- **Somewhat less time than an IBD nurse**
- **About the same amount of time as an IBD nurse**
- **Somewhat more time than an IBD nurse**
- **Much more time than an IBD nurse**

1. Do you feel you have adequate time to discuss each of the following with your UC patients?

| Less than adequate | Adequate | More than adequate | I do not discuss this with patients |
| --- | --- | --- | --- |

1. All symptoms that occurred since the last visit
2. The impact of UC on their lives
3. Questions they have regarding alternative treatment options for their UC
4. Difficulties with 5-ASA adherence/compliance
5. To what extent do you closely probe your patients on whether or not they have any difficulty with taking their 5-ASA medication, even if patients do not mention it up-front?

Select one.

- **Very frequently**
- **Somewhat frequently**
- **Not very frequently**
- **Not at all frequently**

1. The following two questions are somewhat unusual - and very hypothetical in nature - but we would appreciate it if you would give them some thought and indicate your closest feeling...

Firstly, consider a trade-off between UC symptoms and lifespan. What percentage of their remaining lifespan do you think your patients would be willing to forego if they were able to live the rest of their life completely free of UC symptoms?

Please adjust the pointer on the scale below to indicate your opinion.

***Percentage of remaining lifespan willing to forego:***

­­­­­­­­­­­­None------------------------------------------------------------------------------------------------------------50%

1. Now, consider a trade-off between taking UC medication and symptoms. What level of symptoms do you think your UC patients would tolerate in order to take significantly less medication?

Please adjust the pointer on the scale below to indicate your opinion.

***Level of symptoms would tolerate:***

None at all-----------------------------------------------------------------------------------------Very extensive

**SECTION 7 – Patient knowledge and information resources**

1. We realise it can vary significantly from patient to patient, but **in general** how informed do you feel patients are about UC **medications**?

Select one.

- Very informed
- Somewhat informed
- Not very informed
- Not at all informed

1. How helpful do you feel each of the following would be for patients with UC?

| Not at all helpful | Not very helpful | Somewhat helpful | Very helpful |
| --- | --- | --- | --- |

1. Flare rating systems to help them assess their need for immediate care
2. Symptom diaries – to help them recall past flares at check-ups
3. Coping strategies for patients in everyday situations, for example: dealing with UC in the workplace, dating, in college
4. Counseling resources
5. ‘Buddy’ systems to help patients talk with other patients like themselves
6. Educational materials that explain remission and the reasons patients need to be adherent/compliant
7. Tools to help patients stay compliant

**DEMOGRAPHICS**

- 1. In which of the following areas or regions of the country are you currently practising?

Select one.

**GERMANY**

- Brandenburg
- Berlin
- Bayern
- Baden-Württemberg
- Bremen
- Hessen
- Hamburg
- Mecklenburg-Vorpommern
- Niedersachsen
- Nordrhein-Westfalen
- Rheinland-Pfalz
- Sachsen-Anhalt
- Schleswig-Holstein
- Sachsen
- Thüringen

**CANADA**

- British Columbia
- Prairies
- Ontario
- Québec
- Atlantic Provinces
- North

**SPAIN**

- Madrid
- Cataluña
- Valencia / Murcia / Castilla La Mancha
- Andalucia
- País Vasco / Aragón / Navarra
- Galicia / Asturias
- Castilla y León / Cantabria / Extremadura

**UK**

**ENGLAND**

- Yorkshire & Humber
- North East
- North West
- London
- West Midlands
- East Midlands
- East Anglia
- South East
- South West
- Greater London
- Isle Of Man
- Channel Islands

**WALES**

- Wales

**SCOTLAND**

- Scotland

**NORTHERN IRELAND**

- Northern Ireland

**REPUBLIC OF IRELAND**

- Leinster
- Munster
- Connaught
- Ulster

D2. Are you **mostly** office or hospital based?

Select one.

- Office based
- Hospital based
- Equally office and hospital based

D3. How would you describe the location of the main hospital or office in which you work?

Select one.

- City centre
- Suburban
- Rural

D4. Is this a teaching or a non-teaching hospital?

Select one.

- Teaching
- Non-teaching

D5. And is this a public or a private hospital?

Select one.

- Public
- Private

D6. How many other physicians share the same office with you?

[ ] Physicians

D7. Are you:

Select one.

- Male
- Female

D8. Would you mind being contacted again next year to potentially take part in a follow-up survey?

Select one.

1. Yes
2. No
