# Supplementary material for: Perception gaps between patients with ulcerative colitis and healthcare professionals: an online survey
Source: BMC Gastroenterol. 2012 Aug 15;12:108. doi: 10.1186/1471-230X-12-108 (PMC3523079; doi:10.1186/1471-230X-12-108)
Supplement: Additional file 3 — Final questionnaire completed by patients. [file 1471-230X-12-108-S3.doc]

**Appendix 3. Survey of Patients With Ulcerative Colitis**

**SCREENING**

1. In what year were you born?

[ ]

1. Have you been diagnosed as having any of the following conditions?

Select all that apply.

- Diabetes
- Epilepsy
- Asthma
- Heart disease
- Depression
- Arthritis
- Migraine
- Chronic back pain
- Inflammatory bowel disease (IBD), including ulcerative colitis or Crohn’s
- Osteoporosis
- None of these

1. For which of these chronic conditions do you currently take medicine that has been prescribed by a doctor?

*Select all that apply.*

- Diabetes
- Epilepsy
- Asthma
- Heart disease
- Depression
- Arthritis
- Migraine
- Chronic back pain
- Inflammatory bowel disease (IBD), including ulcerative colitis or Crohn’s
- Osteoporosis
- Other chronic conditions
- I do not take prescribed medication for any chronic condition

1. In terms of inflammatory bowel disease (IBD), what is your medical diagnosis?

Select all that apply.

- Ulcerative colitis
- Crohn’s disease
- Indeterminate colitis
- Irritable bowel syndrome (IBS)
- Something else
- Not sure

1. Have you had surgical removal of your colon, or part of your colon?

- Yes
- No

**SECTION 1 – Disease status**

1. What type of healthcare professional would you say provides the **majority** of medical care for your UC?

Select one.

- Hospital consultant / Gastroenterologist
- Gastroenterologist
- Internal medicine physician / Internist
- GP / Family doctor
- Colorectal surgeon
- Nurse specialist
- Other (specify:_______________________________________)

1. In what year were you first diagnosed with UC by a medical doctor?
2. How has your doctor described or classified the severity of your UC overall (not just when you are having a flare-up)?

Select one.

- Mild
- Moderate
- Severe
- Not sure

1. Regardless of how your doctor has described the severity of your UC overall, how would **you personally** describe it?

Select one.

- Mild
- Moderate
- Severe

1. About how many ‘flare-ups’ of your UC have you experienced during the past 12 months?
2. flare-ups
3. How many of these flare-ups did you discuss with your doctor/nurse?
4. flare-ups
5. Why didn't you discuss [all]/[any] of these flare-ups with your doctor/nurse?

Please type in your answer as fully as possible in the box below.

1. Based on your own experience, how would you describe a **typical** flare-up of your UC?

Please type in your answer as fully as possible in the box below.

1. Please rate each of the following to best describe a **typical** ‘flare-up’ of your UC?

Please adjust the pointer on each of the 3 scales below to indicate how you define a **typical UC flare** in respect to frequency of stools per day, frequency of blood in stools and feeling of urgency (to get to the bathroom)

**Stools per day:** 0­­­­­­­­­­­­­­­­­­­------------------5----------------------10---------------------15--------------------20

**Blood in stools:** No stools------------------------------------------------------------------------All stools

**Feeling of urgency:** Weak----------------------------------------------------------------------------Strong

**(to get to the bathroom)**

1. Which of the following best describes your typical response to a flare-up?

Select one.

- I contact a doctor [or nurse]
- I only contact my doctor [or nurse] in certain circumstances, such as a particularly bad flare-up
- I manage alone, without seeing my doctor [or nurse]

1. Based on your own viewpoint, please place in rank order the four causes of UC flare shown below.

Please enter a number between 1 and 4 in each box, with 1 being the most common cause and 4 being the least common.

1. The natural course of the condition
2. Changes from regular diet
3. Stress
4. Not taking preventative therapy when UC is in remission
5. How many times, if ever, have you been hospitalised because of your UC in an emergency situation (not a routine hospital consultation or for endoscopy)?
6. times
7. When your UC is not active, how often do you think about when you will experience your next flare-up?

Select one.

- Almost constantly ­– it is always on my mind
- Multiple times a day
- Once a day
- Once a week
- Once a month
- Less than once a month
- Never

1. Please describe ‘remission’, as you personally experience it?

Please type in your answer as fully as possible in the box below.

1. And which one of these statements **best** describes what remission has realistically meant for you, personally?

Select one.

- Experiencing no symptoms, feeling similar to how I did before I developed UC
- Living with some symptoms, but managing life without interruption
- Living with symptoms and interruptions to daily life, but with less severity, pain and bleeding as during a flare-up
- None of these

1. Again thinking personally, which level of each of the following do you **typically** experience in remission?

Please adjust the pointer on each of the 3 scales below to best reflect your **UC when in remission**

**Stools per day:** 0­­­­­­­­­­­­­­­­­­­--------1--------2-------3--------4--------5--------6--------7--------8--------9------10

**Blood in stools:** No stools-------------------------------------------------------------------------All stools

**Feeling of urgency:** Weak----------------------------------------------------------------------------Strong

**(to get to the bathroom)**

**SECTION 2 – Perception of condition normals, disease control and remission**

1. You mentioned earlier that during the past 12 months you have experienced about ***<<from Q5>>*** flare-ups. Do you consider this number of flare-ups to be “normal” to you, or not normal?

Select one.

- Normal
- Not normal

1. How many flare-ups per year would you consider to be “normal” to you?
2. flare-ups
3. Which of the following three options best describes your UC right now?

Select one.

- Flaring
- Mildly symptomatic/grumbling – fluctuating mild symptoms that can be bothersome but not strong enough to be called a flare-up
- In remission

1. Which one of these statements **best** describes how effectively your UC has been controlled over the past 12 months?

Select one.

- My symptoms were completely or mostly under control
- My symptoms were present but did not interfere with my quality of life
- My symptoms caused some disruption to my quality of life
- My symptoms affected my quality of life on a regular basis

**SECTION 3 – Living with ulcerative colitis**

1. Overall, how disruptive has having UC been to your life?

Select one.

- Very disruptive
- Somewhat disruptive
- Not very disruptive
- Not at all disruptive

1. How disruptive, if at all, is UC when it comes to:

| Not at all disruptive | Not very disruptive | Somewhat disruptive | Very disruptive | Not sure / not applicable |
| --- | --- | --- | --- | --- |

- 1. Your relationship with your spouse or partner
  2. Your relationship with other family and friends
  3. Limiting travel and holidays/vacations
  4. Day-to-day activities
  5. Social activities
  6. Participating in physical activities/sports
  7. Your emotional wellbeing
  8. Your studies, job or career

1. Thinking about the last 12 months (one year), how many days of work and/or college/university have you missed specifically because of your UC?

*Please give your best estimate, even if you’re not completely sure.*

1. days of work missed

- does not apply

1. days of college/university missed

- does not apply

1. To what extent do you agree or disagree with each of the following statements?

| Strongly disagree | Somewhat disagree | Somewhat agree | Strongly agree |
| --- | --- | --- | --- |

1. UC makes my life more stressful
2. UC makes it difficult to lead a normal life
3. Having UC is embarrassing
4. I worry about the long-term health effects of having UC
5. How often do you feel:

| Never | Rarely | Sometimes | Often |
| --- | --- | --- | --- |

1. Reluctant to tell people you have UC
2. Empowered to self-manage and control your UC
3. Depressed about having UC
4. Which one of the following bothers you **the most** about your UC?

Select one.

- Blood in your stools
- Number of stools per day
- Urgency
- Pain
- Number of tablets to be taken
- None of the above

The next question is unusual and is not in any way meant to imply that UC shortens lifespan, or that there is any medication in development that can offer permanent remission. Your response will help build a picture of patients’ view of living with UC. Please try to answer it as honestly as you can.

1. If there were a medication that enabled you live the rest of your life completely free of UC symptoms, but which would reduce your life-expectancy, what percentage of your lifespan would you be willing to forego?

Please adjust the pointer on the scale below to indicate your opinion.

***Percentage of remaining lifespan willing to forego:***

­­­­­­­­­­­­None---------------------------------------------------------------------------------------------50%

1. Similarly, what level of UC symptoms would you be prepared to tolerate in order to take significantly less medication?

Please adjust the pointer on the scale below to indicate your opinion.

***Level of symptoms would tolerate:***

None at all--------------------------------------------------------------------------------------Very extensive

**SECTION 4 – Medications and Adherence**

1. Are you currently taking **prescription** medication for your UC?

Select one.

- Yes
- No, but I’m supposed to
- No

1. Have you ever previously taken aminosalicylate or 5-ASA medication?

To help you remember, types or brands of aminosalicylate/5-ASA medications include:

- - - - mesalazine (Asacol, Pentasa, Salofalk, Claversal, Mezavant, Lixacol, Mesren, Ipocol, Huma-Col-Asa, Novo-5ASA EST)
      - balsalazide (Colazide)
      - sulfasalazine (Salazopyrin, Sulazine, Sulfazalazine)
      - olsalazine (Dipentum)

Select one.

- Yes
- No
- Unsure

1. Which types of medication are you currently being prescribed?

Select all that apply.

- Aminosalicylates/5-ASAs, including:
  - - - mesalazine (Asacol, Pentasa, Salofalk, Claversal, Mezavant, Lixacol, Mesren, Ipocol, Huma-Col-Asa, Novo-5ASA EST)
      - balsalazide (Colazide)
      - sulfasalazine (Salazopyrin, Sulazine, Sulfazalazine)
      - olsalazine (Dipentum)
      - other
- Corticosteroids, including:
  - - - beclometasone (Clipper)
      - prednisone, prednisolone, methylprednisolone (Predenema, Predfoam, Predsol)
      - cortisone, hydrocortisone (Colifoam)
      - budesonide (Budenofalk, Entocort)
      - other
- Immunomodulators or immunosuppressants, including:
  - - - azathioprine (Imuran)
      - cyclosporin A (Deximune, Sandimmun, Neoral)
      - mercaptopurine, 6-MP (Purinethol)
      - sirolimus (Rapamune)
      - tacrolimus (Advagraf, Prograf)
      - other
- Antibiotics, including:
  - - - metronidazole (Flagyl)
      - ciprofloxacin (Cipro, Ciproxin)
      - other
- Biologic therapy, including:
  - - - Remicade (infliximab)
      - Humira (adalimumab)
      - other
- Other type of medication (specify:_____________________________________)
- Not sure

1. How are you currently taking your 5-ASA medication.

Select one.

- Orally/by mouth (tablet, capsule or sachet)
- Rectally (foam, enema or suppository)
- Both orally and rectally

The next set of questions is specifically about the 5-ASA medication that you are currently taking **orally/by mouth** for your UC.

1. How many times per day has your doctor recommended you take your 5-ASA medication when your UC is **flaring-up**?

Select one.

- Once per day
- Twice per day
- Three times per day
- More than three times per day
- Not sure

1. How many times per day has your doctor recommended you take your 5-ASA medication when **in remission**?

Select one.

- Once per day
- Twice per day
- Three times per day
- More than three times per day
- Not sure

1. How difficult, if at all, is it for you to take your 5-ASA medication every day, at the correct time?

Select one.

- Very difficult
- Somewhat difficult
- Not very difficult
- Not at all difficult

1. Thinking about the way in which you took your 5-ASA medication during your **most recent** period of remission; which of the following best describes how you did so?

Select one.

- I tried taking fewer tablets than prescribed (but did not stop taking the medication)
- I tried not taking medication at all for a period of time
- I always took most or all of my medication that my doctor wanted me to

1. Have you mentioned to your doctor that you took less of your 5-ASA medication during this period of remission?

Select one.

- Yes, I have mentioned to my doctor that I took less, but not revealed the extent to which I took less
- Yes, I have mentioned to my doctor that I took less, and the extent to which I took less
- No, I have not mentioned to my doctor that I took less

1. Think about the **last seven days** (one week). Please indicate how much of the prescribed amount of your 5-ASA medication you have taken:

Please adjust the pointer on the scale below to indicate how much of your medication you have taken in the last week

**5-ASA medication:** None­­­­­­ of it ----------------------------------Half of it-------------------------------------All of it

1. And in general, over the **past 12 months**, would you say that you have taken your 5-ASA medication:

Select one.

- Every day
- Most days, but have probably missed several days in total
- Have missed many days in total
- Have missed more days than days I have taken it

1. What are the **main** reasons for missing your 5-ASA medication or not taking it every day?

Select a maximum of two answers.

- I try to cut back on medication when I can
- To avoid possible side-effects
- I forgot to take it
- Have been feeling well
- I find it a burden to take all the medication
- Don’t like to be reminded of my UC
- Other reason [specify:________________________________________________________]

1. Overall, how satisfied/happy are you with your current 5-ASA medication?

Select one.

- Very satisfied/happy
- Somewhat satisfied/happy
- Not very satisfied/happy
- Not at all satisfied/happy

1. Have you discussed your dissatisfaction with your doctor?

Select one.

- Yes
- No

1. As you may or may not know, there are now 5-ASA medications for UC designed to be taken just **once-a-day**, even during flare-ups. Before now, were you aware of these once-a-day medications?

Select one.

- Yes, currently taking a medication like that (where my doctor told me to take only once-a-day – even during flare-ups)
- Yes, familiar with them, but not currently taking
- No, not familiar until now
- Not sure

1. When you have a flare-up of your UC, which of the following treatment actions, if any, would you take **without** first consulting your doctor?

Select all that apply.

- Increase the dose of your oral 5-ASA
- Start taking your oral 5-ASA (if you had stopped taking it during remission)
- Start taking a temporary course of a rectal 5-ASA (suppository)
- Start taking a temporary course of an oral steroid
- None of the above

1. Think about how you feel towards your 5-ASA medication. To what extent do you **agree** or **disagree** with each of the following statements?

| Strongly disagree | Somewhat disagree | Somewhat agree | Strongly agree |
| --- | --- | --- | --- |

1. A double-edged sword that helps control symptoms but I think might have side effects
2. A burden that curtails my freedom, as I have to remember to take it all the time
3. A friend I can rely upon, stopping symptoms when they start to occur or keeping them from happening
4. Would you switch from your current 5-ASA to another one, when in remission, if your doctor suggested you try one designed to be taken **once daily – even during flare-ups**?

Select one.

1. Yes – I would be open to switching after discussing it with my doctor
2. No – I’m happy/doing fine with my current 5-ASA

**SECTION 5 – The Doctor-Patient relationship**

1. Are you currently on a waiting list to see a hospital consultant/gastroenterologist about your UC?

Select one.

- Yes
- No

1. For how long have you been waiting?

Select one.

- Less than 1 month
- 1-2 months
- 3-4 months
- 5-6 months
- 7-9 months
- 10-11 months
- A year or more

1. When did you **last** see a hospital consultant/gastroenterologist about your UC?

Select one.

- Less than 1 month ago
- 1-2 months ago
- 3-6 months ago
- 7-11 months ago
- 1-2 years ago
- 3-5 years ago
- Over 5 years ago
- Can’t remember

1. When do you typically go to see the doctor or specialist nurse who provides the majority of medical care for your UC?

Select one.

- I arrange regular visits
- Only when I'm not feeling well or have a flare-up
- I try to stay away from seeing the doctor/specialist nurse unless a serious flare-up happens
- I never go to see the doctor/specialist nurse

1. Do you feel that the doctor or specialist nurse who provides the majority of the medical care for your UC has enough time for you?

Select one.

- Always
- Often
- Sometimes
- Never

1. Previous research has shown that some people do not always tell their doctor everything about their UC. Which of the following would you say **best** describes your degree of ‘openness’ with your doctor?

Select one.

- I’m completely open, volunteering all information about my UC symptoms and issues
- I’m open but only if my doctor carefully questions me
- I keep some things from my doctor
- I often keep things from my doctor

1. How involved are you in decisions about treating your UC?

Select one.

- The doctors I see make most decisions regarding treatment
- I have an equal partnership with the doctors I see, where we make decisions together
- The doctors I see advise me and help me to understand my options so I can make the best decisions on my own behalf

1. Please indicate how often you believe each of the following statements apply:

| Never | Sometimes | Often | Always |
| --- | --- | --- | --- |

1. I am reluctant to tell my doctor that I am having flare-ups
2. I am reluctant to tell my doctor about the full impact of UC on my life
3. My doctor underestimates how difficult it is to live with UC
4. My doctor is more easily satisfied than I am that my UC is under control
5. My doctor cares about how UC affects my life
6. How would you rate your hospital consultant/gastroenterologist on each of the following?

| Poor | Fair | Good | Excellent |
| --- | --- | --- | --- |

- 1. Accessibility (ease for you to visit him/her)
  2. Approachability (making you feel comfortable so you can properly open-up)
  3. Concern for your emotional needs
  4. Giving explanations in a way that you can understand
  5. Their understanding of how UC impacts your life
  6. Time available to discuss everything at the consultation, without feeling rushed

1. Have you ever taken the initiative and asked your doctor about new medications and treatment options for UC?

Select one.

- Yes
- No

1. When did you **last** ask your doctor about new medications and treatment options?

Select one.

- Less than 1 month ago
- 1-2 months ago
- 3-5 months ago
- 6-11 months ago
- 1-2 years ago
- 3-5 years ago
- Over 5 years ago
- Can’t recall

1. Why have you **not** asked your doctor about new medications and treatment options?

Select all that apply.

- I did not know any new UC treatments were available
- It is my doctor’s role to introduce new treatments to me
- I did not want to irritate or risk upsetting my doctor
- It never occurred to me to ask
- None of these

**SECTION 6 – Information seeking**

1. In general, how **informed** do you feel about the different medications available to treat UC?

Select one.

- Very informed
- Somewhat informed
- Not very informed
- Not at all informed

1. How often do you seek out information about UC and/or the various treatment options that are available?

Select one.

- About once-a-week
- About once-a-month
- About once every few months
- About once-a-year
- Less frequently
- Never

1. What are your **top three** sources of information about UC and the different treatment options available?

Select up to three.

- Doctors/nurses at your hospital
- Doctors/nurses at your GP surgery/practice
- Pharmacists
- UC or healthcare websites on the Internet
- Patient advocacy groups or other support groups (e.g. *insert relevant name of national patient organisation for country*)
- UC social networking sites (e.g., a UC Facebook group, HealingWell.com)
- UC or healthcare blogs
- Attending events sponsored by UC associations or my GP surgery/practice/hospital
- UC-specific newsletters or magazines
- The general media (e.g., TV, magazines, newspapers)
- Family or friends
- Other (specify:_____________________________)
- None of the above

1. How helpful would each of the following be for you?

| Not at all helpful | Not very helpful | Somewhat helpful | Very helpful |
| --- | --- | --- | --- |

1. Information to help you rate the severity of your flare-ups to help you assess your need for immediate care
2. Diaries to record symptoms and flare-ups to help you talk with doctors/nurses at check-ups
3. Tips and strategies to cope with your condition in everyday situations, for example: school, the workplace, dating
4. New ways to talk with other people with UC
5. Materials that explain where UC is in the body
6. Information that explains what remission is and the importance of taking medication as prescribed
7. Tools to remind you to take your medication
8. Information on the long-term risks associated with UC
9. Have you ever done any of the following?

Select all that apply.

- Talked about living with UC, **in-person or over-the-phone**, with someone else who also has the condition
- Shared information about living with UC in **online** discussion groups, chat rooms or on blogs with someone else who also has the condition
- Learned about the experiences of others who also have UC from books, magazines or other **print** media
- Attended a **support group** for patients with UC
- None of the above

**DEMOGRAPHICS**

- 1. Are you:

Select one.

- Male
- Female
  1. What was the highest or last level of school or formal education that you completed?

Select one.

- No formal education
- Primary school only
- Secondary education, but not completed
- Secondary education up to age 16
- Secondary education up to age 18
- Further education: college, vocational, technical, trade, diploma
- Further education: university
- Further education: post-graduate/doctorate/Ph.D
- Don’t know
  1. Which one of the following best describes your current status?

Select one.

- Full-time student
- Retired
- Currently employed on a full-time basis
- Currently employed on a part-time basis
- Not currently employed – but looking for work
- Not currently employed – and not looking for work
- Homemaker (housewife, house-husband)
  1. Which one of the following best describes your current status?

Select one.

- Single
- Have a partner, but not in a serious relationship
- Have a steady partner, but not living with him/her
- Living with a steady partner, but not married
- Married
  1. Are you a member of [*insert relevant name of national patient organisation for country*]?

Select one.

- Yes
- No
  1. Which, if any, of the following would you describe yourself as?

Select all that apply.

- Having a very busy lifestyle
- Having a demanding job
- Having a relatively easy-going lifestyle
- Having an organised life
- None of these
  1. In which of the following areas or regions of the country are you currently living?

Select one.

**GERMANY**

- Brandenburg
- Berlin
- Bayern
- Baden-Württemberg
- Bremen
- Hessen
- Hamburg
- Mecklenburg-Vorpommern
- Niedersachsen
- Nordrhein-Westfalen
- Rheinland-Pfalz
- Sachsen-Anhalt
- Schleswig-Holstein
- Sachsen
- Thüringen

**CANADA**

- British Columbia
- Prairies
- Ontario
- Québec
- Atlantic Provinces
- North

**SPAIN**

- Madrid
- Cataluña
- Valencia / Murcia / Castilla La Mancha
- Andalucia
- País Vasco / Aragón / Navarra
- Galicia / Asturias
- Castilla y León / Cantabria / Extremadura

**UK**

**ENGLAND**

- Yorkshire & Humber
- North East
- London
- West Midlands
- East Midlands
- East Of England
- South East Coast
- South West
- South Central
- North West
- Isle Of Man
- Channel Islands

**WALES**

- South East Wales
- Mid & West Wales
- North Wales
- SCOTLAND
- NORTHERN IRELAND

**REPUBLIC OF IRELAND**

- Leinster
- Munster
- Connaught
- Ulster
  1. Would you mind being contacted again next year to potentially take part in a follow-up survey?

Select one.

1. Yes
2. No
